# Association between bed‐rest time, food intake, and constipation in older nursing home residents

**DOI:** 10.1111/ggi.70025

**Published:** 2025-02-28

**Authors:** Kenta Ushida, Hidetaka Wakabayashi, Shoji Kinoshita, Haruka Tohara, Tokiko Isowa, Kotomi Sakai, Momoko Tohyama, Yuka Shirai, Ryo Momosaki

**Affiliations:** ^1^ Department of Rehabilitation Medicine Mie University Graduate School of Medicine Tsu Japan; ^2^ Department of Rehabilitation Medicine Tokyo Women's Medical University Hospital Tokyo Japan; ^3^ Department of Rehabilitation Medicine Jikei University School of Medicine Tokyo Japan; ^4^ Department of Dysphagia Rehabilitation, Graduate School of Medical and Dental Sciences Tokyo Medical and Dental University Tokyo Japan; ^5^ Department of Nursing, Graduate School of Medicine Mie University Tsu Japan; ^6^ Department of Research Heisei Medical Welfare Group Research Institute Tokyo Japan

**Keywords:** bed rest, constipation, long‐term care insurance, malnutrition, older nursing home residents

## Abstract

**Aim:**

This study aimed to investigate the associations between bed‐rest time and food intake and between bed‐rest time and constipation in older nursing home residents.

**Methods:**

We conducted a cross‐sectional study using data from the Long‐term Care Information System for Evidence (LIFE) database. We used data collected from older nursing home residents registered in the LIFE database between April 2022 and March 2023. We compared outcome data between the short‐bed‐rest group (≥9 h out of bed per day) and the long‐bed‐rest group (<9 h out of bed per day). The outcomes were the percentage of food intake provided in the last 3 days, the percentage of energy sufficiency (the percentage of energy intake to energy requirements), and the incidence of constipation.

**Results:**

The short‐bed‐rest group consisted of 265 people (53.9%). The short‐bed‐rest group showed a significantly higher percentage of food intake (93.1 ± 12.3 vs. 85.2 ± 21.6), a higher percentage of energy sufficiency (104.8 ± 19.4 vs. 92.2 ± 26.2), and a lower incidence of constipation (6.0% vs. 18.5%) than the long bed‐rest group. Multivariable analyses revealed that shorter bed‐rest time was independently and significantly associated with the percentage of food intake (standardized coefficient: 0.28, *P* < 0.001), the percentage of energy sufficiency (standardized coefficient 0.30, *p* < 0.001), and incidence of constipation (odds ratio: 0.12, *P* < 0.001).

**Conclusion:**

Bed‐rest time is associated with food intake and constipation in older nursing home residents. **Geriatr Gerontol Int 2025; 25: 583–587**.

## Graphical abstract

Shorter bed‐rest time is linked to higher food intake and energy sufficiency, and lower constipation rate in older nursing home residents.

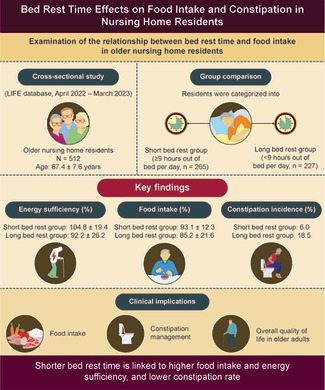

## Introduction

Older nursing home residents are more predisposed to the adverse effects of excessive bed rest, so it is important to reduce their bed‐rest duration. Unnecessary bed rest in older adults decreases their muscle strength, ability to walk, and cardiopulmonary function.^1^ A postural change from supine to sitting is one preventive intervention against the adverse effects of bed rest. In Japan, long‐term care approaches reduce the amount of time spent in bed rest per day to mitigate the adverse effects of this activity on older adults.

Unnecessary bed rest in older adults predisposes them to malnutrition and constipation. In a systematic review, poor activities of daily living (ADL) and physical performance were among the determinants of malnutrition in older adults.^2^ Malnutrition is associated with increased disability, prolonged hospital stays, increased mortality, and increased medical costs.^3^ For the older population, it has been reported that one of the risk factors for constipation is low physical activity levels.^4^, ^5^ Approximately 50%–74% of older adults in nursing homes use laxatives daily.^6^ Physical activity in older adults stimulates appetite, increases energy intake,^7^ and is associated with a lower incidence of constipation.^8^

It is unclear whether the immobility caused by bed rest in older nursing home residents is associated with malnutrition and constipation because most previous studies have focused on people with independent ADL levels.^5^, ^9^ In Japan, 68.7% of older nursing home residents have <60 points on the Barthel Index, and many of them are not independent regarding ADL.^10^ People with a Barthel Index <60 have a severe or worse degree of dependence on the help of another person.^11^ In Europe and China, more than half of the older adults in nursing homes require assistance or are dependent on regarding ADL.^12^, ^13^ We found no studies investigating the association between immobility, malnutrition, and constipation in older nursing home residents, including people with limited ADL, and further evidence is needed to support strategies to reduce bed‐rest time to improve malnutrition and constipation. We hypothesized that bed‐rest time is associated with food intake and constipation in older nursing home residents.

This study aimed to investigate the associations between bed‐rest time and food intake and between bed‐rest time and constipation in older nursing home residents using the Long‐term Care Information System for Evidence (LIFE) database.

## Methods

We conducted this cross‐sectional study using data from the LIFE database, whose details have been reported elsewhere.^14^, ^15^, ^16^ The Japanese Ministry of Health, Labor and Welfare launched a long‐term care insurance services database known as LIFE in April 2021. This database stores information on diseases, physical and cognitive functions, rehabilitation goals and interventions, ADL, instrumental ADL, and nutrition. By using the LIFE database, service providers can charge an additional fee within the long‐term care insurance system. It is expected that the analysis of LIFE data can help improve the business management of care services, guide policy, and increase scientific knowledge. The LIFE data used in this study were stored in the electronic medical record system of Care Connect Japan. This study used cross‐sectional data from 748 nursing home residents enrolled in the LIFE database between April 2022 and March 2023, which were obtained after obtaining consent from the long‐term care facilities. This study was approved by the Ethics Committee of Mie University (H2022‐210) and was conducted as per the ethical standards of the 1964 Declaration of Helsinki and its subsequent amendments.

### Participants

For the 748 participants from eight facilities using the electronic medical record system of Care Connect Japan, we extracted data from the dataset that had no missing data on bed‐rest time. Almost all participants from two of the eight facilities had missing data on bed‐rest time. We excluded those who were unable to have any food intake.

### Exposure and outcome

We compared outcome data between the short‐bed‐rest group and the long‐bed‐rest group. We defined the short‐bed‐rest as spending at least 9 h out of bed. Older nursing home residents spend an average of 9 h per day sitting.^17^ The short‐bed‐rest group is more active than the long‐bed‐rest group.

We evaluated the percentage of food intake provided, the percentage of energy sufficiency, and the incidence of constipation as outcomes. The percentage of food intake refers to how much the older nursing home residents consumed from the main and side dishes within the last 3 days. We defined the percentage of energy sufficiency as the product of the amount of energy supplied and the percentage of food intake divided by the amount of energy required, multiplied by 100. The required nutrient energy contained in the LIFE database is calculated by the facility's nutritionist based on the Dietary Reference Intakes for Japanese.^18^ Recent cases of constipation were the focus of the evaluation of the incidence of constipation.

### Statistical analysis

Categorical data were presented as frequencies and percentages and compared using the χ^2^‐test. Continuous data were presented as the mean ± standard deviation and compared using the *t*‐test. We then conducted logistic regression analyses to analyze the outcomes using a forced entry method.

The covariates adjusted for were the following variables: age, body mass index (BMI), sex, degree of independence in the daily life of elderly with dementia (DIDLED), Vitality Index, Barthel Index, percentage of food intake provided, and incidence of constipation. DIDLED has categories in order of severity of dementia: independent, I, IIa, IIb, IIIa, IIIb, IV, and M.^19^ The Barthel Index is used to assess an individual's ADL, with a higher Barthel Index score indicating greater independence in ADL.^20^ The Vitality Index consists of five subscales related to common basic activities, with higher scores indicating greater motivation.^21^ We also included the following variables in baseline analyses to characterize the older nursing home residents: requiring long‐term care, required nutrients energy, provided nutrients energy, need for dysphagia diet, laxative use, daily sitting time, number of times the person stood up per day, time spent outside of the person's room per day, number of times the person engaged in hobbies and role activities per week. The “requiring long‐term care” variable is a classification from Japan's public long‐term care insurance system based on the need for care, and those classified as requiring long‐term care level 5 (RLTC5) are those with the most serious health issues.^22^

Statistical analyses were performed using SPSS software (version 25.0; IBM Japan, Tokyo, Japan). The threshold for statistical significance was set at *P* < 0.05.

## Results

Among the 512 people with no missing data on bed‐rest time, we excluded 20 people who did not have any food intake. We included the remaining 492 people (mean age: 87.4 ± 7.6 years, females: 76.0%).

Table 1 shows the sociodemographic features of our study participants. The short‐bed‐rest group was made up of 265 people (53.9%). In the baseline analysis, people in the short‐bed‐rest group had the following characteristics compared with those in the long‐bed‐rest group: a higher mean Vitality Index (6.7 ± 2.4 vs. 4.4 ± 2.7), a higher mean Barthel Index (46.3 ± 24.5 vs. 23.0 ± 22.4), a lower percentage of RLTC5 (12.1% vs. 37.4%), more provided nutrients energy (1430.2 ± 188.2 vs. 1385.0 ± 266.8), less need for dysphagia diets (51.7% vs. 68.7%), less laxative use (18.5% vs. 35.2%), longer sitting time per day (11.2 ± 2.1 h vs. 5.4 ± 2.2 h), more number of times standing up per day (15.2 ± 10.2 vs. 6.9 ± 7.5), more time spent outside of the person's own room per day (10.0 ± 3.3 h vs. 5.2 ± 2.5 h), more number of times of hobby and role activities per week (3.9 ± 3.4 vs. 3.2 ± 3.0).

**Table 1:** Baseline sociodemographic data

	Short‐bed‐rest group	Long‐bed‐rest group	*P*‐value
Number of patients, *n*	265	227	
Age, years, mean ± SD	87.2 ± 7.5	87.7 ± 7.7	0.486
Body mass index, mean ± SD	21.1 ± 11.9	21.8 ± 19.6	0.650
Females, *n* (%)	200 (75.5)	174 (76.7)	0.832
Degree of Independence in the Daily Life of Elderly with Dementia, *n* (%)			
Independent	5 (2.2)	7 (2.6)	1.000
I	7 (3.1)	7 (2.6)	0.792
II a	5 (2.2)	18 (6.8)	0.018
II b	33 (14.5)	48 (18.1)	0.330
III a	70 (30.8)	107 (40.4)	0.030
III b	39 (17.2)	36 (13.6)	0.314
IV	57 (25.1)	31 (11.7)	<0.001
M	11 (4.8)	10 (3.9)	0.656
Vitality Index, mean ± SD	6.7 ± 2.4	4.4 ± 2.7	<0.001
Barthel Index, mean ± SD	46.3 ± 24.5	23.0 ± 22.4	<0.001
Requiring long‐term care, *n* (%)			
1	2 (0.8)	1 (0.4)	1.000
2	15 (5.7)	7 (3.1)	0.194
3	134 (50.6)	53 (23.3)	<0.001
4	82 (30.9)	81 (35.7)	0.291
5	32 (12.1)	85 (37.4)	<0.001
Required nutrients energy, mean ± SD	1287.3 ± 219.0	1275.4 ± 284.8	0.603
Provided nutrients energy, mean ± SD	1430.2 ± 188.2	1385.0 ± 266.8	0.029
Required nutrients energy (per kilogram of actual body weight), mean ± SD	28.4 ± 3.6	28.9 ± 5.5	0.194
Provided nutrients energy (per kilogram of actual body weight), mean ± SD	31.0 ± 5.7	31.0 ± 7.8	0.113
Need for dysphagia diet, *n* (%)	137 (51.7)	156 (68.7)	<0.001
Laxative use, *n* (%)	49 (18.5)	80 (35.2)	<0.001
Sitting time per day, mean ± SD	11.2 ± 2.1	5.4 ± 2.2	<0.001
Number of times standing up per day, mean ± SD	15.2 ± 10.2	6.9 ± 7.5	<0.001
Time spent outside of room per day, mean ± SD	10.0 ± 3.3	5.2 ± 2.5	<0.001
Number of times hobbies and role activities per week, mean ± SD	3.9 ± 3.4	3.2 ± 3.0	0.013

Abbreviation: SD, standard deviation.

Table 2 shows the percentage of food intake provided, the percentage of energy sufficiency, and the incidence of constipation. The short‐bed‐rest group had the following features compared with the long‐bed‐rest group: higher percentage of food intake provided (93.1 ± 12.3 vs. 85.2 ± 21.6, *P* < 0.001), higher percentage of energy sufficiency (104.8 ± 19.4 vs. 92.2 ± 26.2, *P* < 0.001), and lower incidence of constipation (6.0% vs. 18.5%, *P* < 0.001).

**Table 2:** Comparison of outcomes between the two groups

	Short‐bed‐rest group	Long‐bed‐rest group	*P*‐value
Percentage of food intake provided, mean ± SD	93.1 ± 12.3	85.2 ± 21.6	<0.001
Percentage of energy sufficiency, mean ± SD	104.8 ± 19.4	92.2 ± 26.2	<0.001
Incidence of constipation, *n* (%)	16 (6.0)	42 (18.5)	<0.001

Abbreviation: SD, standard deviation.

Tables 3 and 4 show the results of the multivariable analysis for bed‐rest time. Shorter bed‐rest time was significantly associated with the percentage of food intake provided (standardized coefficient: 0.28, *P* < 0.001), the percentage of energy sufficiency (standardized coefficient: 0.30, *P* < 0.001), and the incidence of constipation (odds ratio: 0.12, *P* < 0.001).

**Table 3:** Association between bed‐rest time, percentage of food intake provided, and energy sufficiency ratio in multiple regression analyses

	Unstandardized coefficient			Standardized coefficient	*P*‐value
	β	95% confidence interval	Standard error		
Percentage of food intake provided	10.77	6.14–15.40	2.35	0.28	<0.001
Percentage of energy sufficiency	15.89	9.75–22.04	3.13	0.30	<0.001

*Note*: The following covariates were adjusted for: age, BMI, sex, Degree of Independence in the Daily Life of Elderly with Dementia, Vitality Index, Barthel Index, and the incidence of constipation.

**Table 4:** Association between bed‐rest time and incidence of constipation in logistic regression analyses

	Odds ratio	95% confidence interval	*P*‐value
Incidence of constipation	0.12	0.04–0.32	<0.001

*Note*: The covariates adjusted for were as follows: age, BMI, sex, Degree of Independence in the Daily Life of Elderly with Dementia, Vitality Index, Barthel Index, and percentage of food intake provided.

## Discussion

In this cross‐sectional study, we investigated the associations between bed‐rest time and food intake and between bed‐rest time and constipation in older nursing home residents using the LIFE database. The results showed that older nursing home residents with shorter bed‐rest times had a higher percentage of food intake provided, a higher percentage of energy sufficiency, and a lower incidence of constipation compared with those who had longer bed‐rest times. To our knowledge, this is the first study to investigate the associations between bed‐rest time and food intake and between bed‐rest time and constipation in older nursing home residents.

The older nursing home residents in the short‐bed‐rest group had a higher food intake compared with those in the long‐bed‐rest group. Previous studies have suggested an association between food intake and higher activity levels in older adults with independent ADL, which is consistent with the results of our study for older nursing home residents.^9^, ^23^ The older nursing home residents in the short‐bed‐rest group in this study spent more time outside of their rooms and engaged in hobbies and role activities more frequently than did those in the long‐bed‐rest group. Physical activity may contribute to maintaining or increasing the appetite of older adults.^24^ Older nursing home residents with shorter bed‐rest times have increased physical activity due to the increased opportunities for activity and participation, which may have led to an increase in food intake.

The older nursing home residents in the short‐bed‐rest group had a lower incidence of constipation than those in the long bed‐rest group. People with prolonged bed rest are more likely to get constipation owing to decreased peristalsis and constrictive sphincters.^25^ The upright posture promotes gas transit owing to changes in hydrostatic force distribution, increased intra‐abdominal pressure, and a propulsive intestinal motor response induced by exercise.^26^, ^27^ The older nursing home residents in the short‐bed‐rest group in this study had longer sitting times and stood up more frequently than did those in the long‐bed‐rest group. Reducing the time spent in the supine posture and increasing that spent in the upright posture may improve intestinal function and lead to a decreased incidence of constipation.

Attempts to reduce bed‐rest time for older nursing home residents may improve food intake and constipation, leading to improved ADL and quality of life (QOL). Malnutrition is associated with decreased ADL and QOL in older adults.^28^, ^29^ Constipation is associated with frailty, psychological impairment, and decreased QOL.^30^ Improvement of constipation may lead to a reduction in medical costs for laxatives. Future studies should use longitudinal data to investigate the cause‐to‐effect relationship between approaches to reducing bed‐rest time, food intake, and constipation in older nursing home residents.

This study had several limitations. First, we excluded older nursing home residents with missing data on bed‐rest time, which may have caused a selection bias. Second, in the multivariable analysis, we did not adjust for all items related to food intake and constipation. For example, the need for assistance with eating and using the toilet, the use of nutritional supplements, and the details of rehabilitation programs. Although these items are recorded in the LIFE database, the inclusion of more items in a multivariable analysis would increase the number of cases excluded owing to missing values. In the future, enough number of cases should be included, and all items related to food intake and constipation should be adjusted for. Third, the LIFE database study has just begun, and we could only obtain cross‐sectional data. Therefore, we were unable to perform a longitudinal study, and the causal relationship was unclear. Future studies should use databases that include longitudinal data. Fourth, the correlation between food intake and constipation may have influenced the results of this study. There is a correlation between insufficient food intake and constipation.^31^ However, the cause‐to‐effect relationship between food intake and constipation is not clear. If future studies investigate this relationship, the association between reduced bed rest, food intake, and constipation may be clarified.

## Conclusion

Our study suggests that shorter bed‐rest times in older nursing home residents contribute to improvements in the percentage of food intake provided, the percentage of energy sufficiency, and the incidence of constipation. If future longitudinal studies clarify the cause‐to‐effect relationship, attempts to reduce bed‐rest time may constitute an effective strategy to improve food intake and constipation in older nursing home residents.

## Disclosure statement

The authors have no relevant financial or nonfinancial conflicts of interest to disclose.

## Author contributions

Conceptualization: Kenta Ushida, Momoko Tohyama, Yuka Shirai. Methodology: Kenta Ushida, Ryo Momosaki. Formal analysis and investigation: Kenta Ushida, Momoko Tohyama, Yuka Shirai. Writing–original draft preparation: Kenta Ushida. Writing–review and editing: Hidetaka Wakabayashi, Haruka Tohara, Tokiko Isowa, Kotomi Sakai, Shoji Kinoshita, Ryo Momosaki. Funding acquisition: Ryo Momosaki. Resources: Ryo Momosaki. Supervision: Hidetaka Wakabayashi, Haruka Tohara, Tokiko Isowa, Kotomi Sakai, Shoji Kinoshita;

## Ethics approval statement

This study was approved by the Ethics Committee of Mie University (H2022‐210) and was conducted as per the ethical standards of the 1964 Declaration of Helsinki and its subsequent amendments.

## Data Availability

The data from this research are available from the corresponding author upon reasonable request.
